# Breast ductography: to do or not to do? A pictorial essay

**DOI:** 10.1186/s13244-023-01547-x

**Published:** 2023-11-23

**Authors:** Afsaneh Alikhassi, Belinda Curpen

**Affiliations:** Breast Imaging Division, Medical Imaging Department, Sunnybrook Health Sciences Centre, University of Toronto, University of Toronto, 2075 Bayview Avenue, M6, Toronto, ON M4N 3M5 Canada

**Keywords:** Ductography, Nipple discharge, Ultrasound, Mammography, MRI

## Abstract

Nipple discharge is a frequent breast disease clinical presentation. Although most cases of nipple discharge are physiologic, pathologic nipple discharge is not uncommon. Eight to 15% of pathological nipple discharge is associated with malignancy, requiring investigation. Some specialists believe that ductography is a challenging procedure that is better to be substituted by other methods, such as MRI. However, an experienced physician can perform ductography quickly and easily and still play an essential role in some clinical scenarios. Conventional imaging, such as mammography and sonography, commonly fails to detect the underlying causes of pathological nipple discharge. MRI has limitations of low specificity, cost, lengthy exam duration, accessibility, and patient factors such as claustrophobia. In addition, we can make a specific diagnosis and appropriate treatment by coupling ductography with other methods, such as ultrasound-guided or stereotactic biopsy. This study aims to present the ductography technique, possible findings, and the clinical settings where ductography is useful.

**Critical relevance statement** Although ductography is currently less used in breast imaging, it still plays an essential role in some clinical scenarios. These clinical scenarios include pathological nipple discharge with negative conventional imaging, contraindicated MRI, unavailable MRI, unremarkable MRI results, and multiple MRI findings.

**Key points**

• Conventional imaging commonly fails to detect the underlying causes of pathological nipple discharge.

• MRI in the setting of nipple discharge has some limitations.

• Ductography still plays an essential role in some clinical scenarios.

• Coupling ductography with other methods helps make a specific diagnosis.

**Graphical Abstract:**

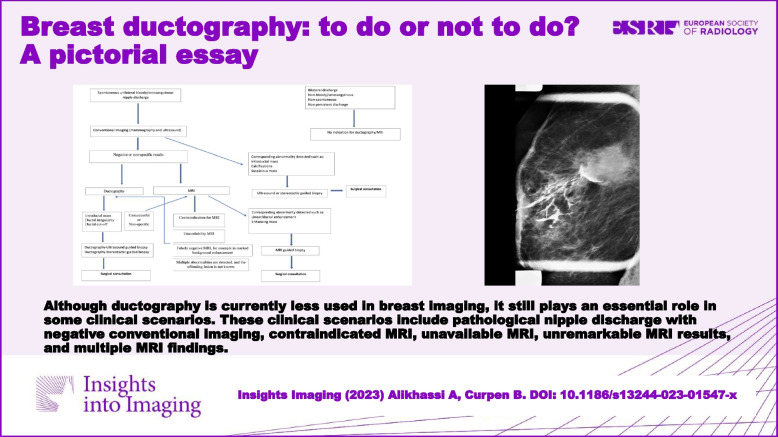

## Introduction

Nipple discharge is a commonly seen presentation in breast clinics and represents 4.8% of the presenting symptoms in all women with breast-related complaints [1]. In most cases, nipple discharges are non-pathological and are not associated with breast neoplasms [2]. Physiological discharges are commonly white, yellow, or green and occur from several ducts bilaterally. The etiology of physiological discharges include hypothyroidism, pituitary prolactinoma, and side effect from medication [3, 4].

Pathological nipple discharge is described as uniorifice, spontaneous, bloody, clear, or serosanguinous. Ductal carcinoma in situ (DCIS) and papillary lesions are the most common etiology for pathological nipple discharge [5]. Since 8–15% of pathological nipple discharges are related to malignant neoplasms, it necessitates investigating [6].

Some specialists believe that ductography is a time-consuming and challenging procedure that can be substituted by other methods, such as sonography or MRI [2, 7, 8]. However, in many cases, conventional imaging, such as mammography and sonography, fails to detect the underlying causes of pathological nipple discharge since the lesions are small and prove to be completely intraductal [6, 9]. Although contrast-enhanced MRI demonstrates high sensitivity for detecting malignancy when there is a pathological discharge, there are some limitations: low specificity, MRI cost, time-consuming exam, availability, gadolinium allergy, and severe claustrophobia [9–14].

If there is persistent pathologic nipple discharge with negative conventional imaging, the surgeon may proceed with duct excision, which is both diagnostical and therapeutic. However, this blind approach may not always identify the exact cause of the discharge and the remaining pathology. Moreover, this technique is not advised for women of child-bearing age. Metanalysis of studies evaluated nipple discharge fluid assessment suggested that cytology also has limited diagnostic accuracy [15]. Currently, in some centers, ductoscopy is used as a minimally invasive diagnostic technique in pathologic nipple discharge with the potential for simultaneous interventions. However, this method has some limitations, such as variable ductal anatomy with limited accessibility of distal smaller ducts, scarred or occluded ducts, acute angulated ducts, false tract formation, cost, and lack of a validated scoring system for ductoscopic visualizations [16, 17].

This study aims to summarize a ductography technique, the possible findings, and clinical settings where ductography is useful. By adding ductography to other methods, we can make a specific diagnosis and treat it appropriately [18].

### Procedure

Before ductography, mammography is done with mediolateral and craniocaudal views to assess any suspicious background findings, such as microcalcifications or masses; we do not try to elicit the discharge before the test. A warm towel can be used on the center of the breast for 10–15 min to relax the periareolar sphincter muscle. The nipple and areola are cleaned and sterilized and then draped. Gentle periareolar pressure must be applied to locate the exact orifice from which the discharge is coming. Expressing too much of the discharge will interfere with identifying the orifice.

A focused light and magnifying lens are used to facilitate cannulating the duct. A small 30-gauge straight blunt-tip cannula is used to inject the ductal system with contrast. We do not routinely use lacrimal dilators in our center. The sterile anesthetic gel can coat the cannula tip and insert it into the pore. Approximately 0.2–0.4 mL of iodinated contrast is injected, and before that, we need to ensure that there is no air bubble in the cannula, syringe, and extension tubing.

Contrast is injected slowly until the patient reports pain, the operator feels resistance, or contrast reflux is seen. Then, the injection needs to be stopped immediately. Immediately after injection, the catheter needs to be fixed on the skin to prevent contrast leakage. The catheter is removed in our department, and collodion is used on the nipple surface as a glue to prevent contrast leakage. Subareolar magnification views in craniocaudal and true lateral projections are the standard mammogram views taken at our institute. This test typically requires about 30 min.

### Summary of ductography steps

- A high-intensity lamp, magnifying lens, and examination bed in a quiet, private room are required.
- The preferred position is an oblique supine position with the ipsilateral arm raised.
- Gentle pressure is used to express the discharge.
- Cannulation of the orifice can be tried.
- Once the duct is cannulated, 0.2–0.3 mL of contrast material is injected.
- We use collodion to prevent the contrast from leaking. A cannula can also be taped in place in preparation for imaging instead.
- Magnification views are obtained in lateral and craniocaudal views.

### Ductography contraindications

Ductography is contraindicated in case of severe allergic reaction to iodinated contrast materials history, non-collaborating patients for any reason, and a previous nipple surgery that would distort the underlying ducts. Other possible contraindications include nipple retraction, which makes cannulation impossible, serious concurrent medical illnesses, and mastitis or breast abscess. Implants and augmentations may obscure ductographic findings [1, 2].

### Ductography findings

The findings can include filling defects in the contrast filled duct, ductal dilatation, ductal irregularity, and abrupt cut-off.

**Fig. 1:**
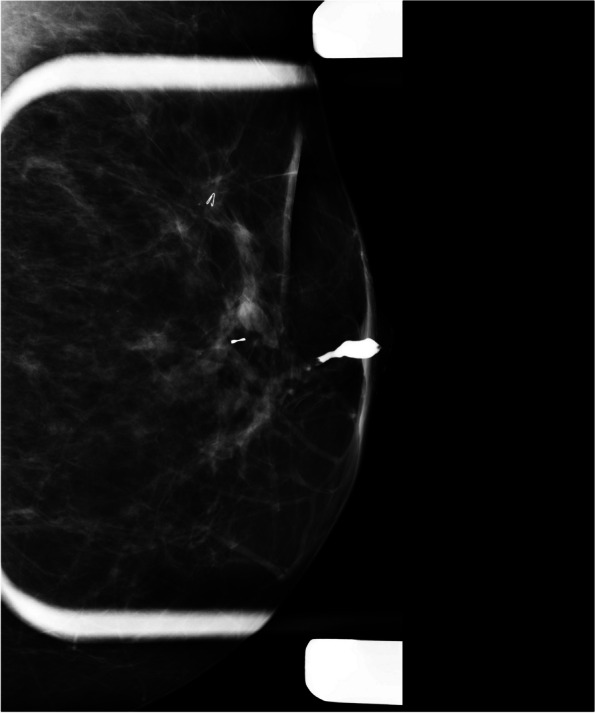
Left breast ductography shows a dilated duct with proximal ductal obstruction

*Complete ductal obstruction* can be observed in both benign and malignant neoplasms. In benign papillary lesions, contrast material mainly outlines the leading edge of lesions partially, resulting in the meniscus appearance. The cut-off site in the carcinoma is often irregular with a moth-eaten appearance (Fig. 1) [19, 20].

**Fig. 2:**
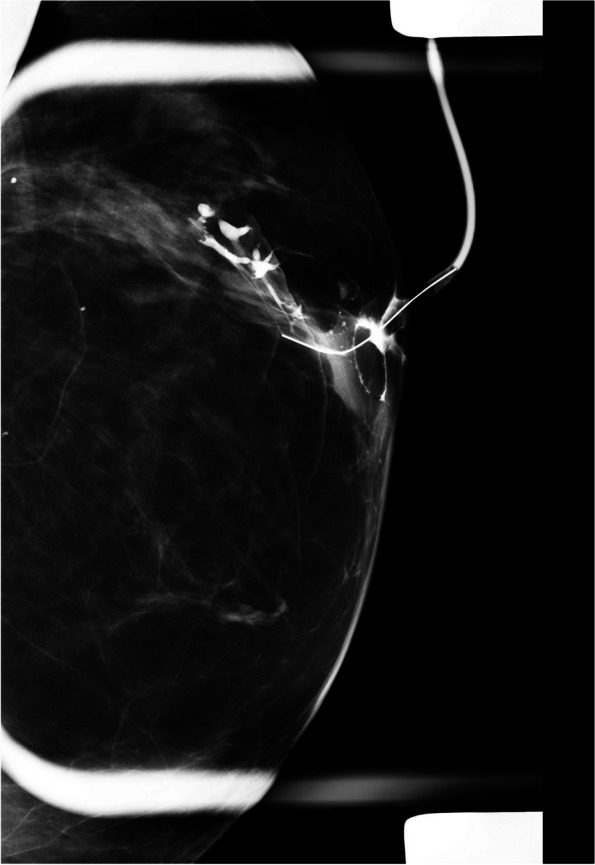
Left breast ductography demonstrates dilated duct with distal irregular filling defect and obstruction

*Ductal wall irregularity* in ductography is nonspecific and may be seen in both benign papillomas and malignant causes. This finding can be extensive or local, making it hard to evaluate thoroughly in conventional imaging, especially when it is peripheral (Fig. 2) [21].

**Fig. 3:**
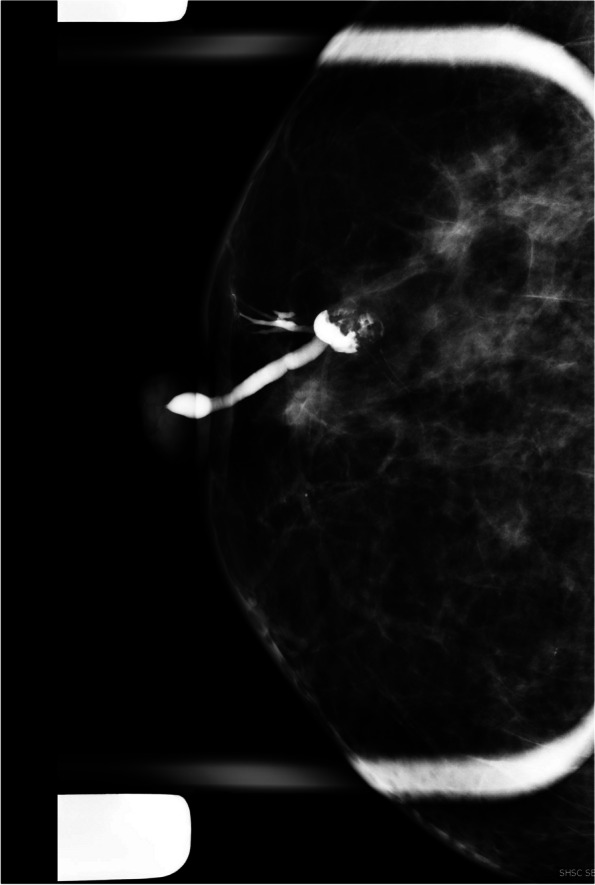
Right breast ductography shows dilated duct with multiple filling defects

*Multiple irregular filling defects*, especially if seen in nondilated peripheral ducts, suggest malignancy. Peripherally located papillomas are seen as multiple intraductal filling defects with smooth or lobular surfaces and are often associated with ductal dilatation (Fig. 3) [6].

*Extravasation *can be a normal finding because forceful contrast material injection causing wall perforation and ductography should be rescheduled within 7–14 days after the initial attempt. Extravasation can be seen with a carcinoma’s destruction of the ductal wall. In that case, the patient does not feel pain during the procedure [6, 21].

**Fig. 4:**
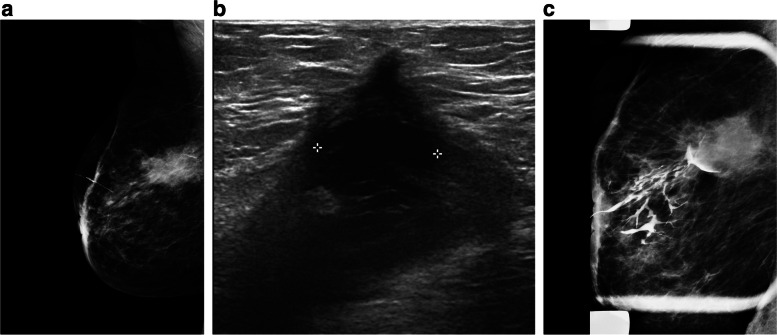
A 82-year-old woman with a history of resected DCIS in the right breast. She presented with a 3-week history of serous sanguineous spontaneous nipple discharge. There were post-surgical changes in the right upper outer quadrant, including the known seroma, which appeared slightly decreased overall compared to previously. The right breast ultrasound did not show any suspicious lesion. In ductography, multiple ducts were opacified and extended into the upper outer breast towards the lumpectomy site with a cut-off at the seroma site. No filling defect within the ducts was identified. The nipple discharge was suspected to be coming from a seroma secondary to fistulization of the seroma within the ducts. A short-term follow-up US was recommended. After a few months, the discharge stopped

*Ductal displacement *is another possible finding in ductography, which is not specific evidence of a benign or malignant space-occupying lesion. Any associated findings, such as mammographic asymmetry, architectural distortion, or sonographic abnormality, are helpful for diagnosis (Fig. 4A–C) [6–22].

**Fig. 5:**
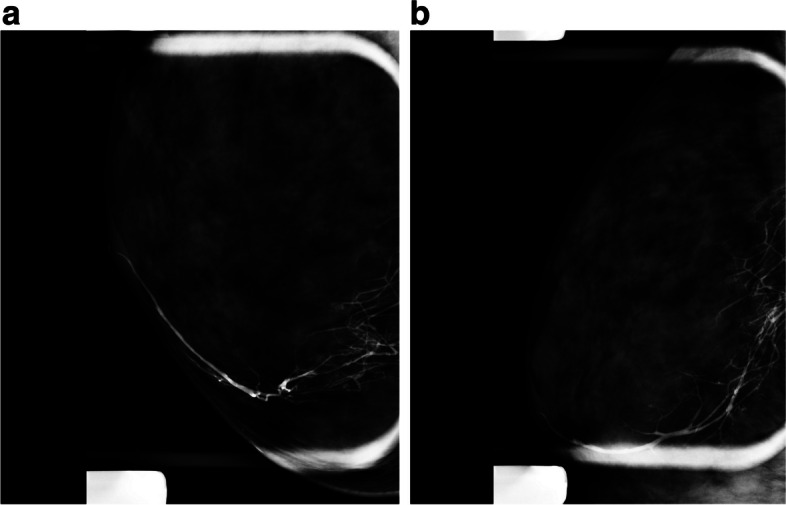
A 48-year-old woman presented with right breast brown color nipple discharge. Ductography radiographs show adequate opacification of the ducts. There is no evidence of a filling defect to suggest the cause of nipple discharge. There are areas of mild ductal dilatation, some anterior pruning, and mild dilation of the terminal ductal lobular unit suggestive of fibrocystic change. The nipple discharge stopped within 6 months

*Ducts communicating with cysts*, communication between ducts and cysts can cause nipple discharge with decompression of cyst fluid into the ducts. Normal or dilated ducts communicating with cysts suggest fibrocystic changes in ductography (Fig. 5A, B) [21].

### Imaging modalities for evaluation of pathological nipple discharge

*Mammography and ultrasound* are indicated for all women with pathological nipple discharge. Calcifications are the most important finding that we look for in mammography. In pathological nipple discharge, mammography is positive in 50–90% of patients with malignancy but less than 50% with benign papillomas [2, 3]. Ultrasound (US) by an experienced sonologist may detect intraductal masses [2]. In standard practice, the US is seldom helpful in establishing the cause of nipple discharge [2]. In the study by Srinivasan, similar sensitivities were reported for ductography and conventional breast imaging in patients with breast cancer, while in high-risk lesions, ductography was significantly more sensitive [14]. In patients with intraductal benign papilloma, ductography was more sensitive than non-invasive imaging, but with a minimal difference. The same study showed that conventional imaging had better specificity in distinguishing benign and malignant lesions [14]. In the recent study by Younjung Choi, ductography was combined with US and shows higher sensitivity (94.1% [16/17]) for detecting malignancy in patients with pathologic nipple discharge and negative mammography than initial ultrasound (47.1% [8/17], *p* = 0.013) [23].

*MRI* with intravenous injection of contrast material has been commonly reported to be highly sensitive for the detection of invasive breast carcinoma, which includes breast cancer in the setting of nipple discharges [24]. Several studies strongly support the benefit of contrast-enhanced breast MRI for evaluating patients with suspicious nipple discharge. A sensitivity of 85.7% to 100% for contrast-enhanced MRI to detect malignancy in this setting was reported [9–13]. Breast MRI with intravenous injection of contrast material can demonstrate the extent of disease and has the potential to distinguish between malignant and benign lesions [9–13]. However, there are some limitations in using breast MRI in the presence of nipple discharge, such as cost, exam length, accessibility, and claustrophobia [5].

*Magnetic resonance ductography (MRD)* is a non-invasive method with heavily T2-weighted sequences without contrast. It shows the dilated ducts as tubular structures with high signal intensity, and intraductal lesions appear as signal defects, duct wall irregularities, or ductal obstructions [10]. Like conventional ductography, differentiation of benign from malignant lesions is impossible, and neither technique can demonstrate the extent of disease in all cases. Fusion imaging with MRD and breast MRI has the potential to show not only intraductal abnormalities but also the extent of lesions [9–13]. Conventional ductography is more readily available, and its cost is less than that of MRD. MRD is contraindicated for some patients with MRI contraindications. MRD does not reveal a duct when it is not dilated, but conventional ductography may show a non-dilated duct after cannulation. In a study by Nicholson et al., MRD’s sensitivity was about 52.6–55.0%, which was not superior to conventional ductography [10].

### Scenarios in which ductography is still useful

**Fig. 6:**
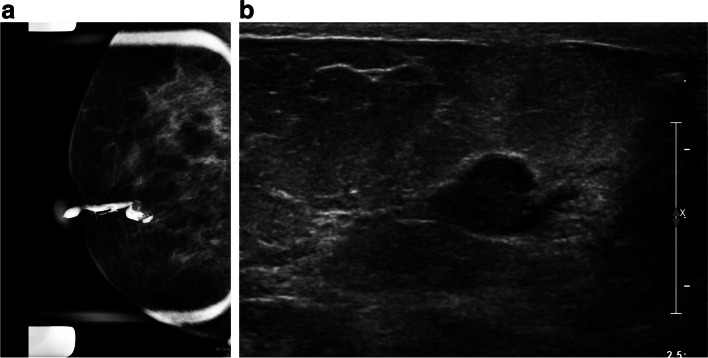
A 43-year-old woman with bloody nipple discharge and several likely benign masses in her US underwent ductography via cannulation of the duct with discharge. Ductography helped to find the pathology. Ultrasound-guided biopsy of the mass showed benign papilloma

**Fig. 7:**
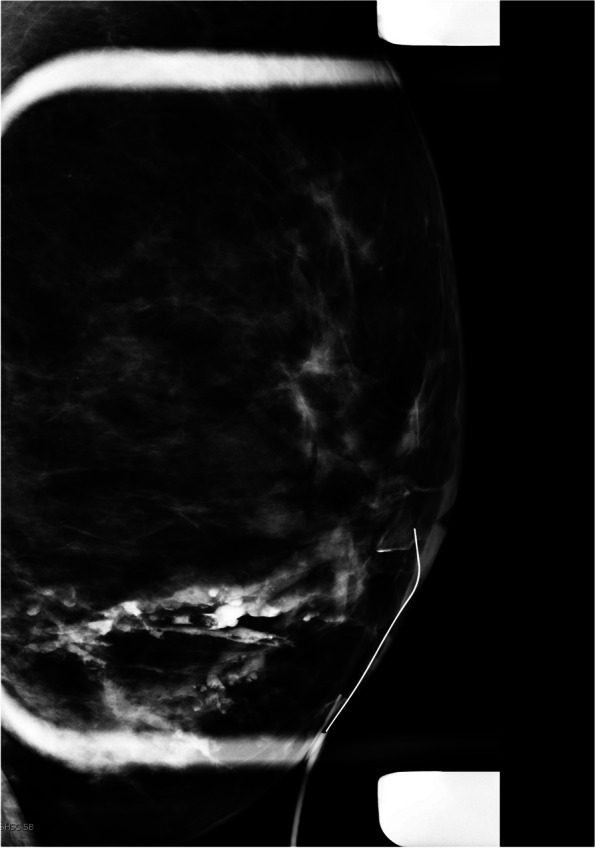
A 52-year-old woman with clinical concern of unilateral spontaneous bloody nipple discharge underwent ductography. Diffuse small intraductal filling defects were due to ductal carcinoma in situ without invasive components in microscopic pathology

**Fig. 8:**
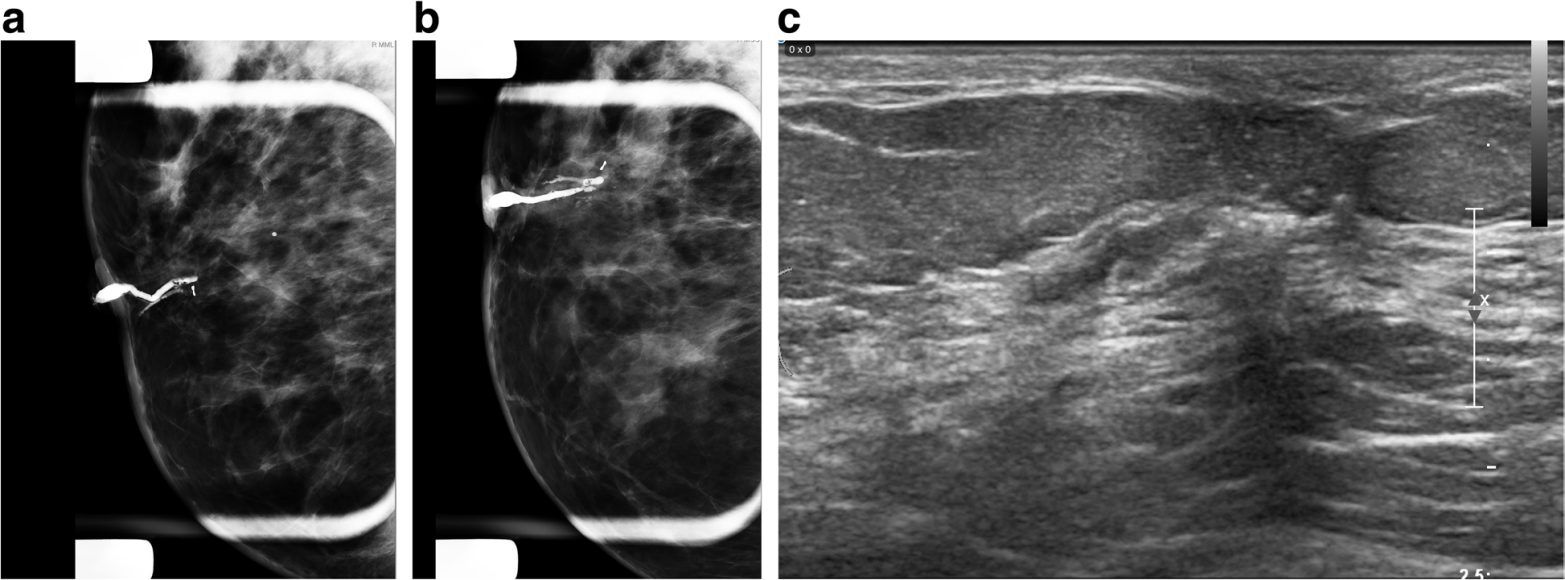
A 50-year-old female presented for evaluation of clear right nipple discharge. Ductography radiographs show an irregular filling defect in the subareolar duct. The post-biopsy marker clip from a previous biopsy was demonstrated adjacent to the site of the filling defect. The patient was returned to the US room. The selected duct within the right breast was distended with contrast and better seen. It appeared to terminate at an isoechoic intraductal mass at the 6:00 subareolar position. A US-guided biopsy of this lesion was performed. The pathology result showed benign papilloma with mild usual ductal hyperplasia and no evidence of atypia

**Fig. 9:**
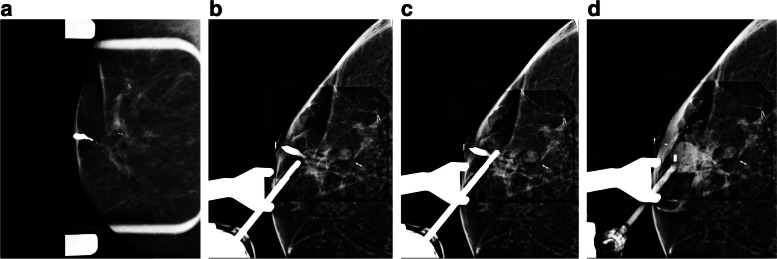
A 63-year-old woman presented with right breast spontaneous bloody nipple discharge with a history of 2 previous benign biopsies in the same breast. Conventional imaging did not show any new findings. Ductography images show a dilated duct with a filling defect. A targeted US immediately after ductography did not detect any abnormality. A ductography-guided stereotactic biopsy was arranged, and a biopsy of the filling defect under stereotactic biopsy was performed. The pathology result was a benign papilloma

1. When a patient has pathological nipple discharge and multiple lesions detected on conventional imaging or MRI, such as multiple papillomas. With ductography, we can find the offending lesion and target it for biopsy or preoperative localization (Fig. 6A, B).
2. Diffuse small intraductal cancers without microscopic invasive components might present with bloody nipple discharge. In these cases, palpable abnormalities are frequently absent, and conventional imaging results such as mammography and US are mostly negative or nonspecific (Fig. 7).
3. Ductography is helpful in pathological nipple discharge with negative conventional imaging and contraindication for MRI or unavailability of MRI.
4. In cases of falsely negative MRI, such as marked background parenchymal enhancement or low-grade DCIS, ductography can show the nature and extent of the lesions [25, 26].
5. When all imaging is negative, and there is persistent ductal discharge if blind central duct excision is performed without ductography, there may be under or over-excision [14, 27, 28]. There is a risk of leaving the underlying lesion behind. It is worthwhile doing the ductography before surgery.
6. With negative conventional images and findings such as filling defects in ductography, we can do a target US immediately after ductography. Dilatation of the duct with contrast facilitates the conspicuity of the lesion in US. US biopsy can then be performed in the same session (Fig. 8A–C).
7. Ductography-guided procedure is sometimes required in cases of persistent pathological nipple discharge and no lesion in conventional images, and the abnormality is detected only with ductography such as ductography-stereotactic biopsy (Fig. 9A–D) [29]. At our center, ductography-biopsy is followed by clip placement. The clip facilitates future preoperative localization in cases requiring surgery.
8. Rarely, surgeons may request preoperative ductography with diluted methylene blue dye iodinated contrast to mark the abnormal discharging duct [30]. This safe and inexpensive technique helps surgeons to determine the precise location and volume of tissue that needs excision.
9. Percutaneous ductography technique can be considered when there is a dilated duct in the US, and conventional ductography is indicated, but the canulation of the orifice is technically impossible. US-guided needle placement is performed with the needle attached to a small-volume syringe filled with contrast material or even methylene blue preoperatively. The contrast material is administered under US guidance. Then, the needle is removed, and standard magnification spot views are obtained.

**Fig. 10:**
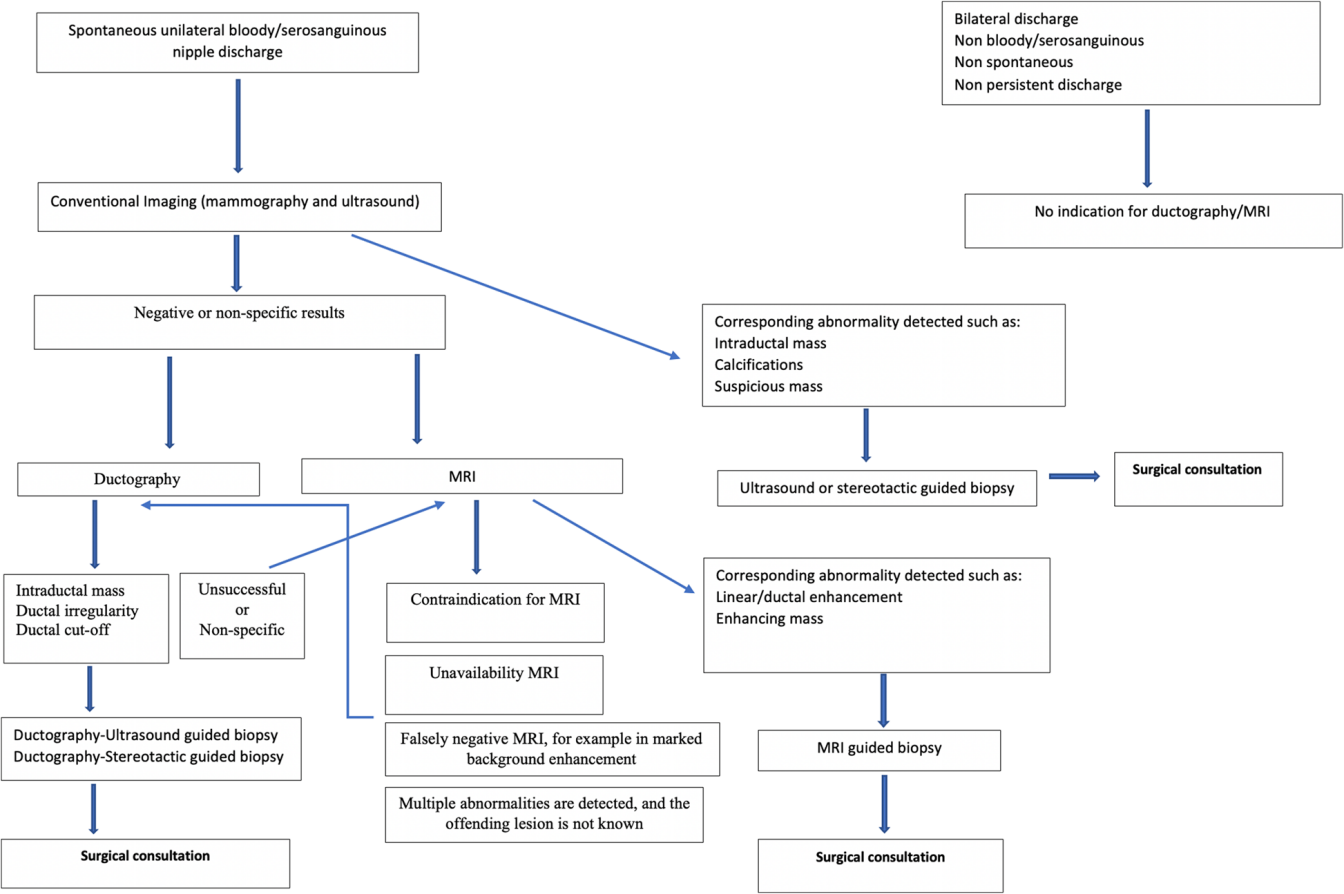
Summary of the imaging approach to pathologic nipple discharge

Figure 10 summarizes the imaging approach to pathologic nipple discharge. Of note, in the setting of pathologic nipple discharge with negative conventional imaging, there is no robust data to show whether we should start with ductography or MRI first. Radiologists can choose either based on the clinical scenario and their internal guidelines. In our center, we perform ductography first unless there is any contraindication for ductography.

### Disadvantages of conventional ductography

Conventional ductography is semi-invasive and can be uncomfortable for patients. Although ductography can be performed quickly and easily by an experienced physician, it is sometimes a challenging and time-consuming procedure for less experienced radiologists. If the discharge is scant or not seen on the day of the procedure, identification and cannulation of the duct is not possible. The incorrect duct may be cannulated sometimes.

Common complications of conventional ductography are duct perforation, extravasation, and rarely mastitis. Hypersensitivity to iodinated contrast material is rare [2]. We have not encountered a contrast material allergy in our practice. In cases of duct perforation and contrast extravasation, the procedure should be immediately stopped and rescheduled for 1–2 weeks later. The vasovagal reaction is not common, and to avoid a vasovagal reaction, we do all our ductography cases in a supine position.

## Summary

Although ductography is currently less used in breast imaging worldwide, it is still beneficial and indicated in some scenarios, such as pathological nipple discharge with negative conventional imaging, contraindicated MRI, unavailable MRI, unremarkable MRI results, and multiple MRI findings. Coupling ductography with US or stereotactic biopsy is very helpful in determining patients who will be surgical candidates.

## Data Availability

Not applicable.

## Abbreviations

DCIS: Ductal carcinoma in situ
MRD: Magnetic resonance ductography
US: Ultrasound
